# Supplementary material for: Mechanistic Insights Revealed by YbtPQ in the Occluded State
Source: Biomolecules. 2024 Mar 8;14(3):322. doi: 10.3390/biom14030322 (PMC10967856; doi:10.3390/biom14030322)
Supplement: Supplementary file 1 [file biomolecules-14-00322-s001.zip › biomolecules-2868477-supplementary.pptx]

## Slide 1
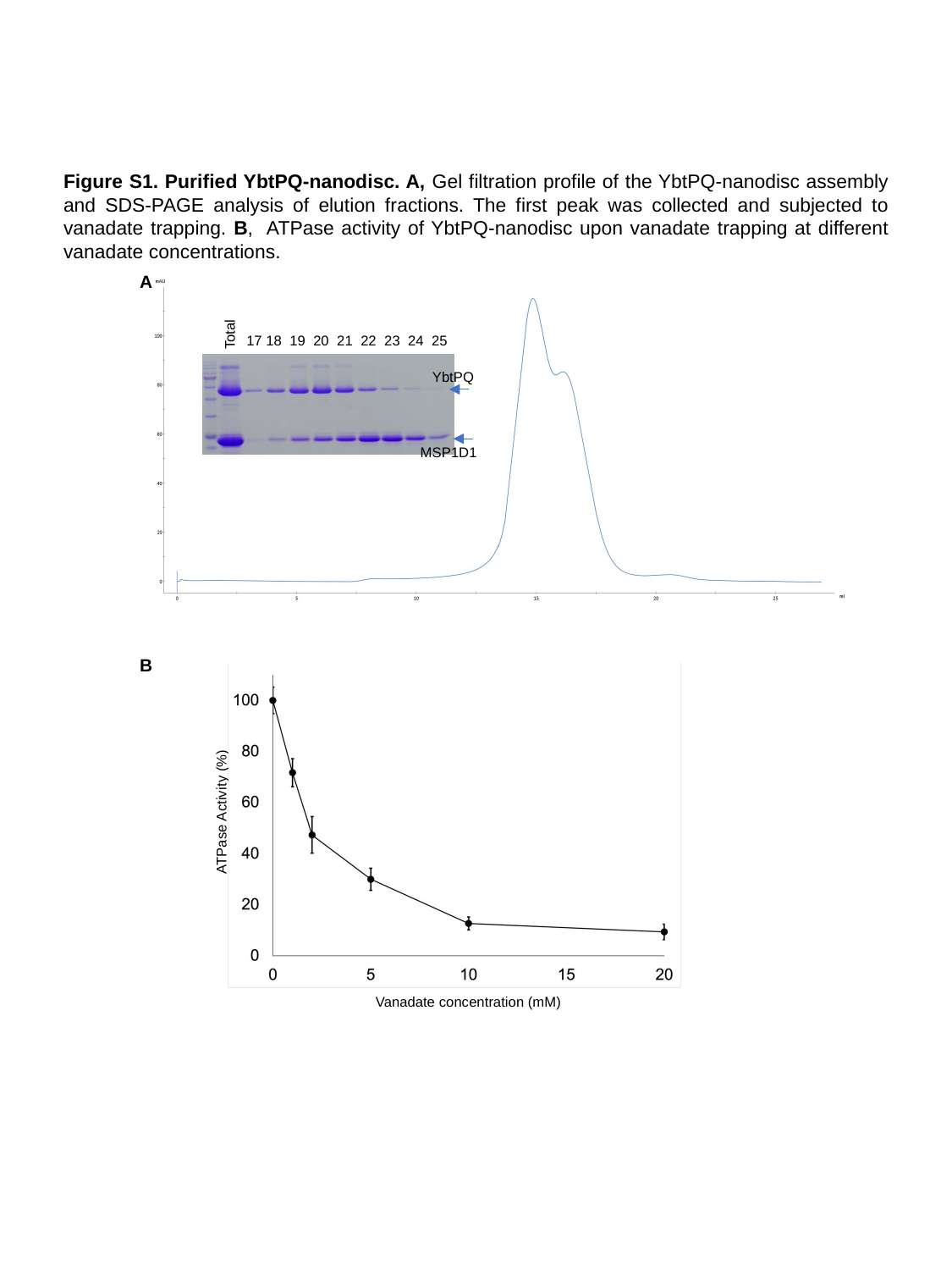

Figure S1. Purified YbtPQ-nanodisc. A, Gel filtration profile of the YbtPQ-nanodisc assembly and SDS-PAGE analysis of elution fractions. The first peak was collected and subjected to vanadate trapping. B, ATPase activity of YbtPQ-nanodisc upon vanadate trapping at different vanadate concentrations.
A
Total
17 18 19 20 21 22 23 24 25
YbtPQ
MSP1D1
B
ATPase Activity (%)
Vanadate concentration (mM)

## Slide 2
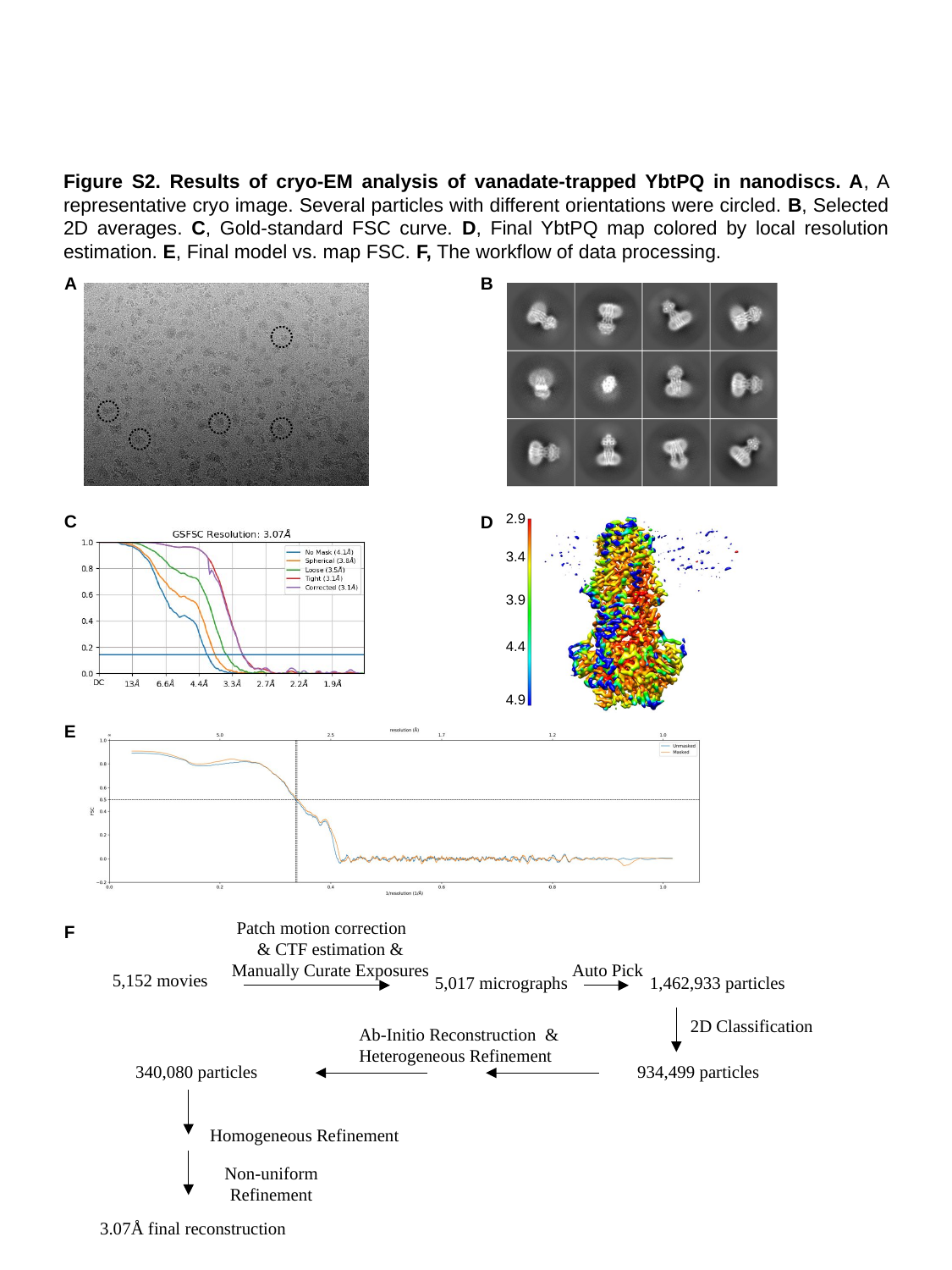

Figure S2. Results of cryo-EM analysis of vanadate-trapped YbtPQ in nanodiscs. A, A representative cryo image. Several particles with different orientations were circled. B, Selected 2D averages. C, Gold-standard FSC curve. D, Final YbtPQ map colored by local resolution estimation. E, Final model vs. map FSC. F, The workflow of data processing.
B
A
2.9
C
D
250Å
3.4
3.9
4.4
4.9
E
Patch motion correction & CTF estimation & Manually Curate Exposures
F
Auto Pick
5,152 movies
5,017 micrographs
1,462,933 particles
2D Classification
Ab-Initio Reconstruction & Heterogeneous Refinement
340,080 particles
934,499 particles
Homogeneous Refinement
Non-uniform Refinement
3.07Å final reconstruction

## Slide 3
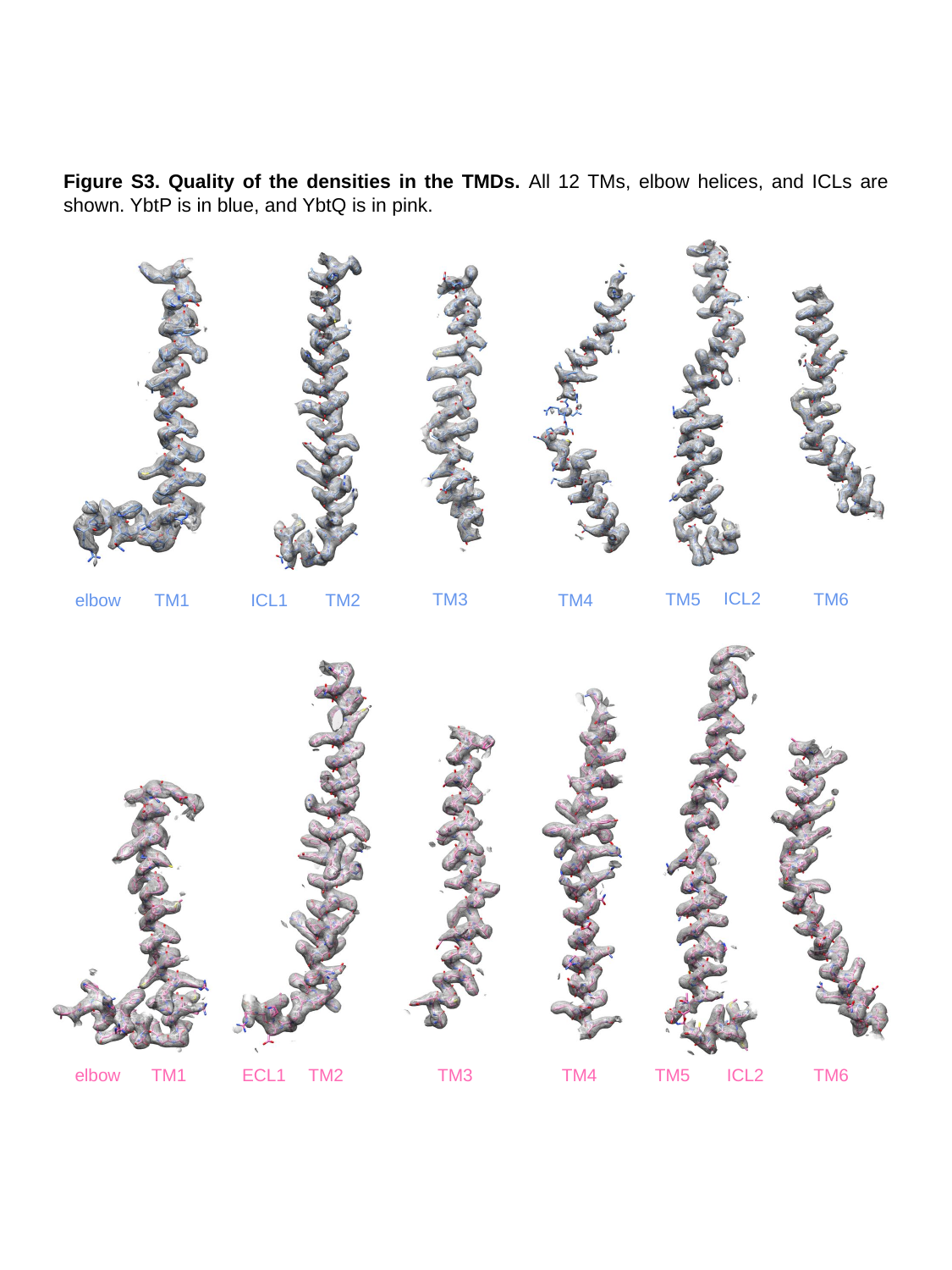

Figure S3. Quality of the densities in the TMDs. All 12 TMs, elbow helices, and ICLs are shown. YbtP is in blue, and YbtQ is in pink.
ICL2
TM3
TM5
TM6
elbow
TM1
ICL1
TM2
TM4
elbow
TM1
ECL1
TM2
TM3
TM4
TM5
ICL2
TM6

## Slide 4
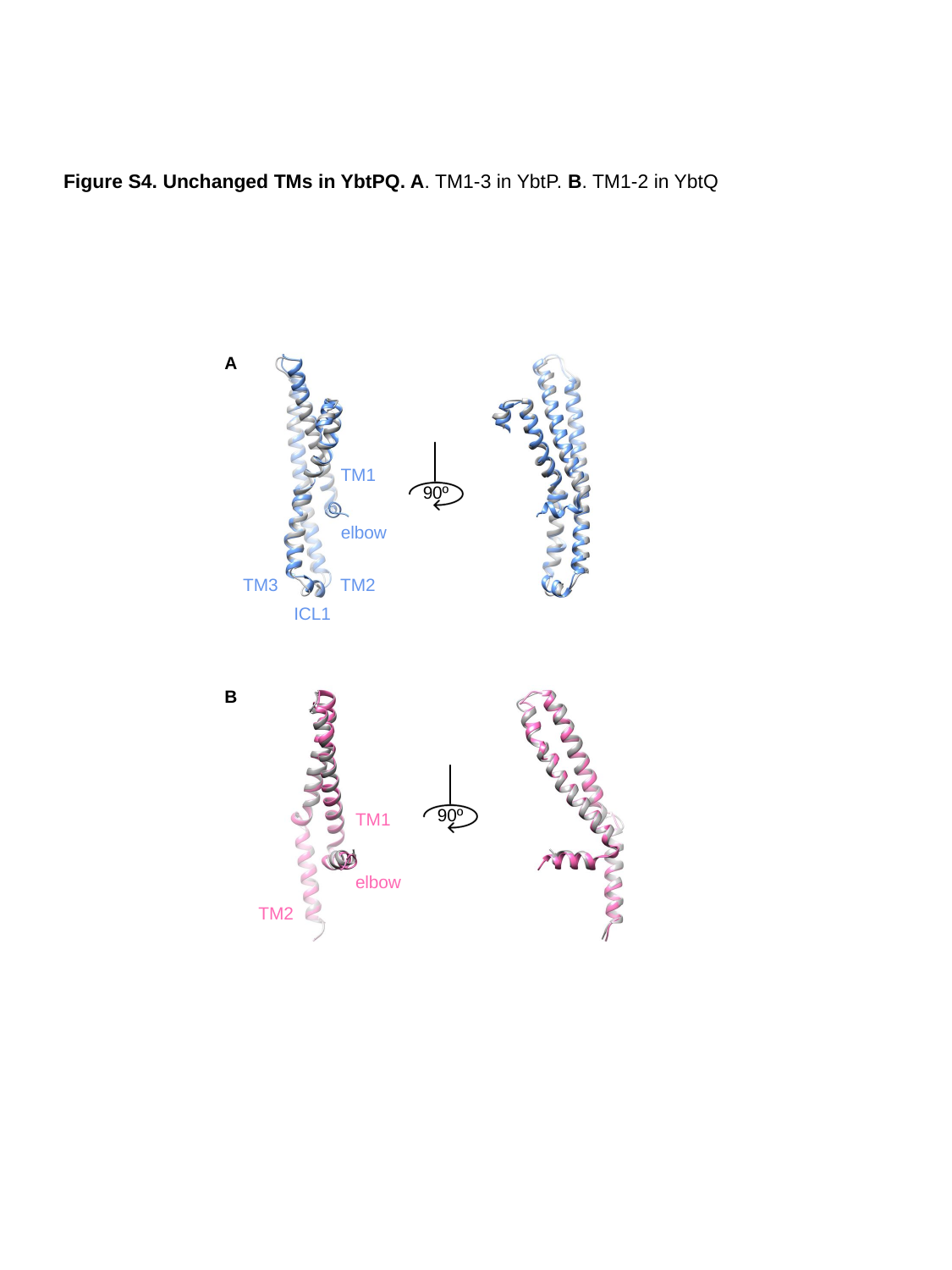

Figure S4. Unchanged TMs in YbtPQ. A. TM1-3 in YbtP. B. TM1-2 in YbtQ
A
TM1
90º
elbow
TM3
TM2
ICL1
B
90º
TM1
elbow
TM2

## Slide 5
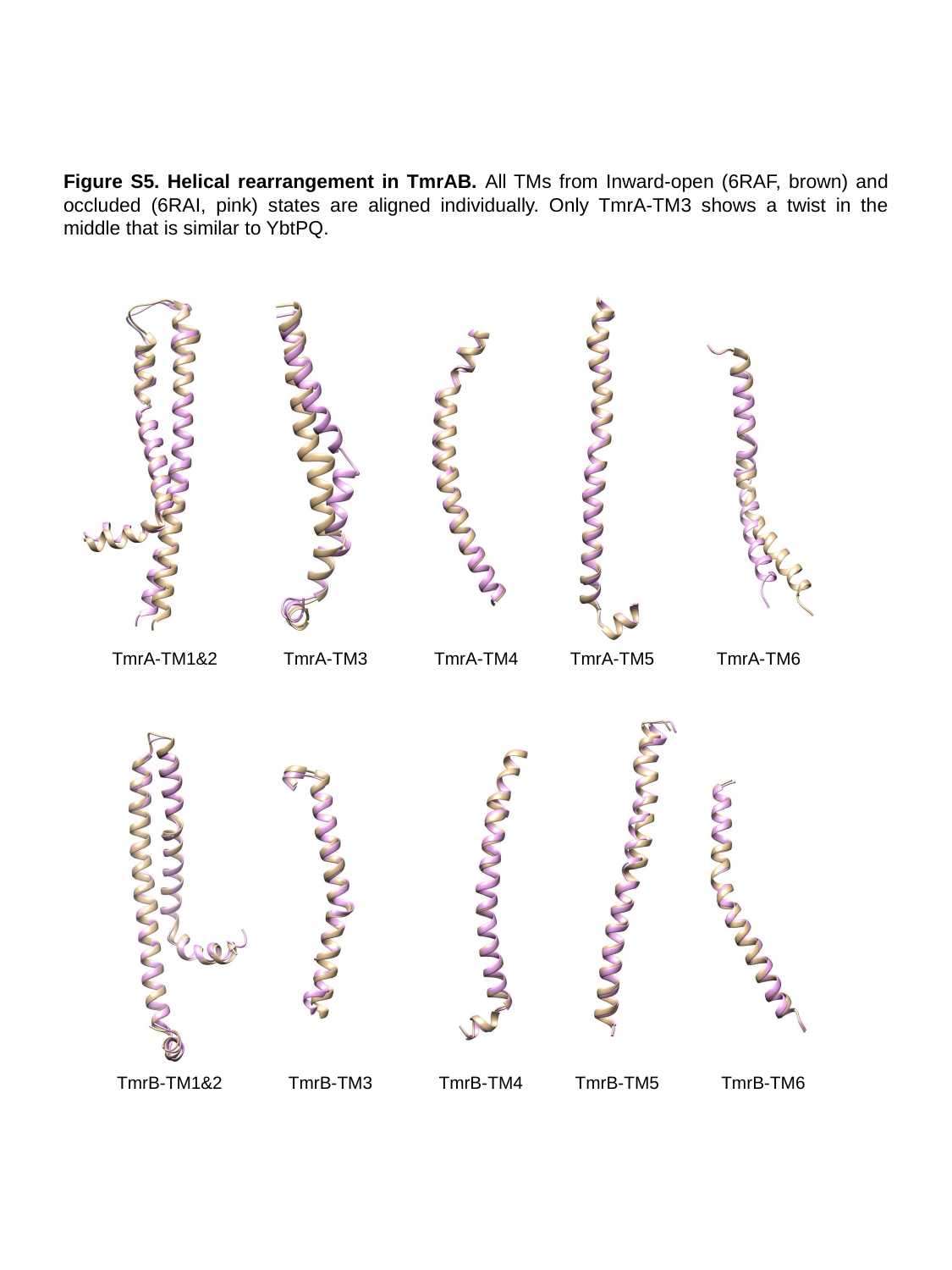

Figure S5. Helical rearrangement in TmrAB. All TMs from Inward-open (6RAF, brown) and occluded (6RAI, pink) states are aligned individually. Only TmrA-TM3 shows a twist in the middle that is similar to YbtPQ.
TmrA-TM3
TmrA-TM4
TmrA-TM5
TmrA-TM6
TmrA-TM1&2
TmrB-TM3
TmrB-TM4
TmrB-TM5
TmrB-TM6
TmrB-TM1&2

## Slide 6
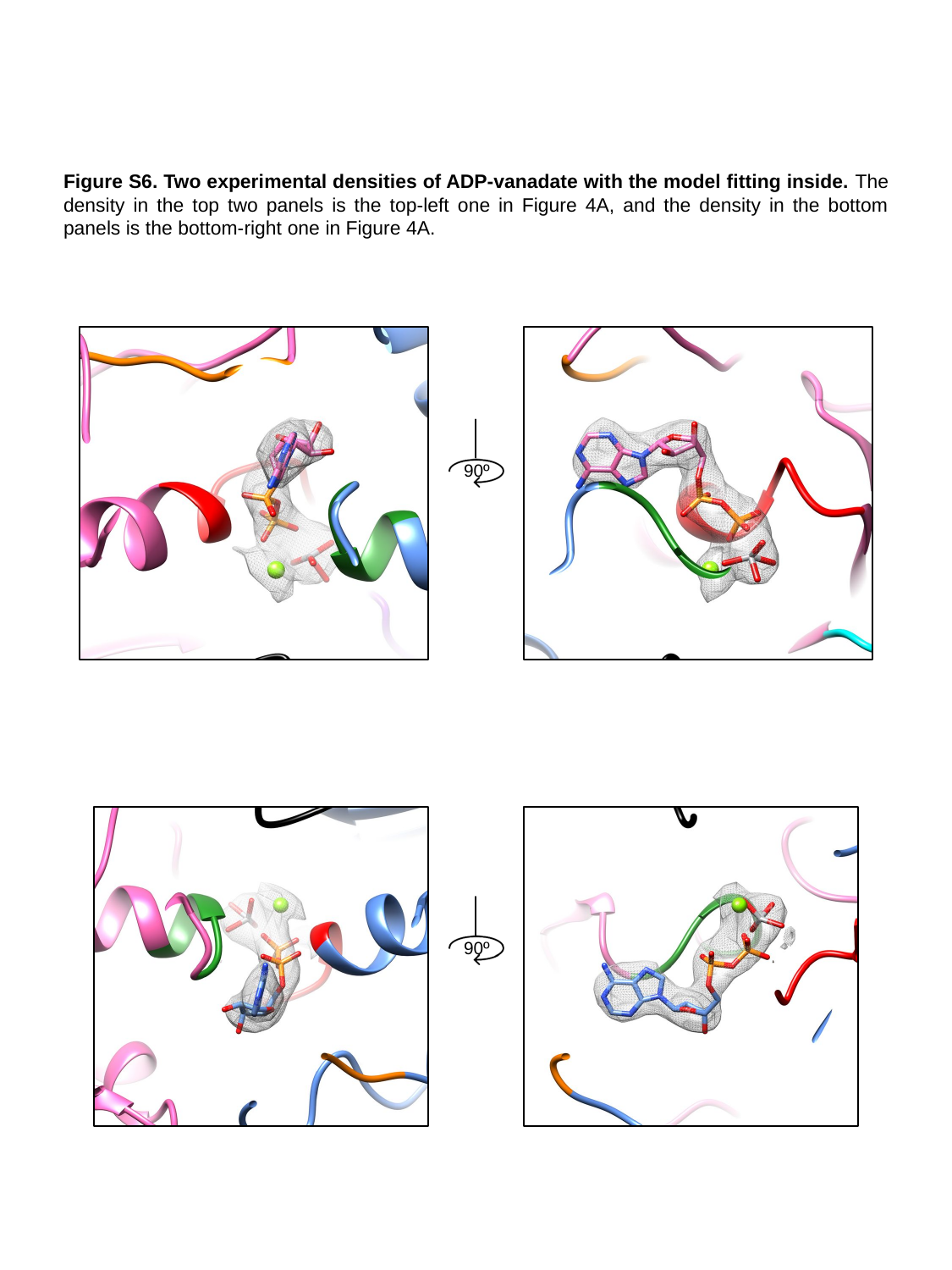

Figure S6. Two experimental densities of ADP-vanadate with the model fitting inside. The density in the top two panels is the top-left one in Figure 4A, and the density in the bottom panels is the bottom-right one in Figure 4A.
90º
90º

## Slide 7
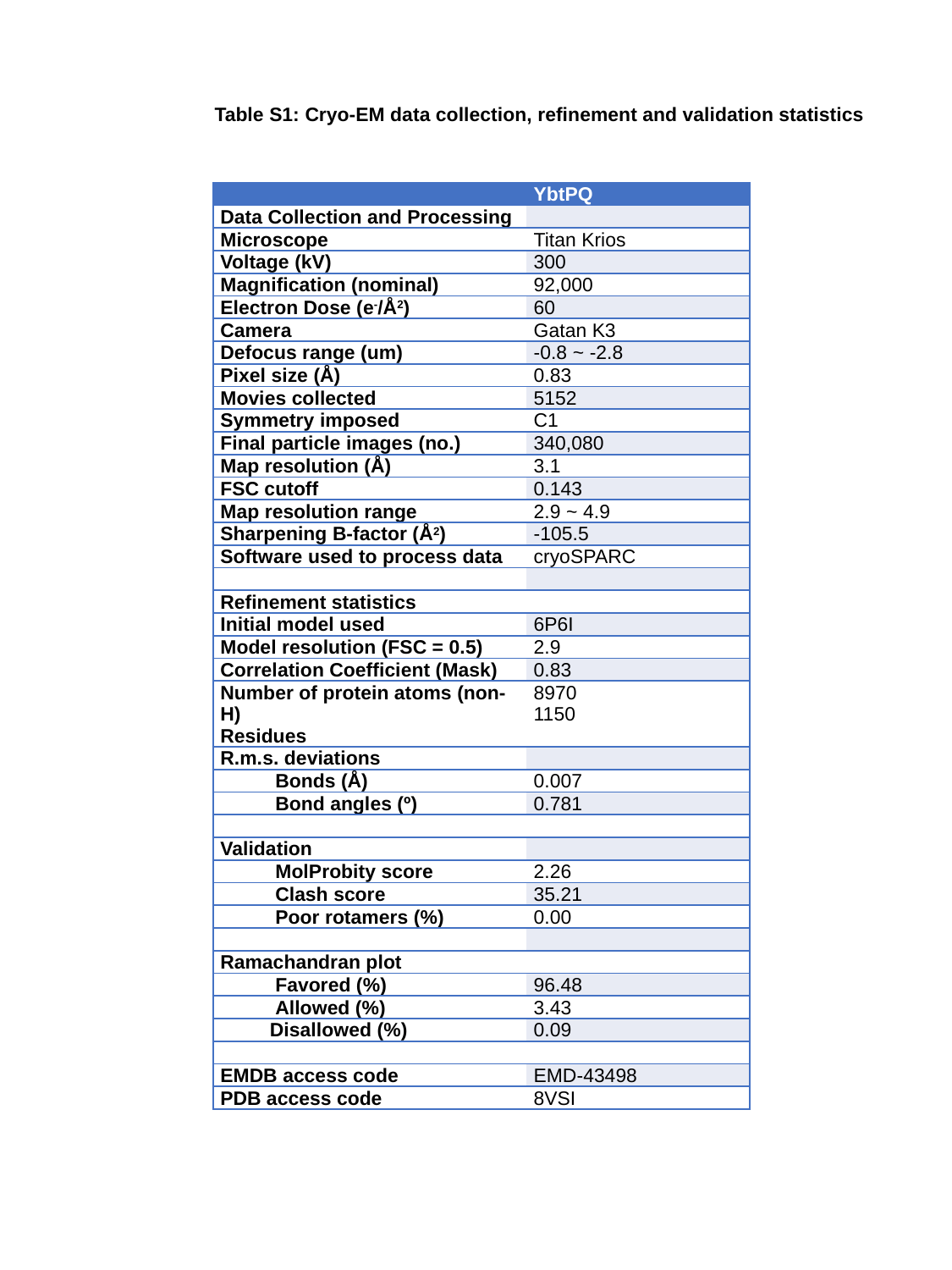

Table S1: Cryo-EM data collection, refinement and validation statistics
| | YbtPQ |
| --- | --- |
| Data Collection and Processing | |
| Microscope | Titan Krios |
| Voltage (kV) | 300 |
| Magnification (nominal) | 92,000 |
| Electron Dose (e-/Å2) | 60 |
| Camera | Gatan K3 |
| Defocus range (um) | -0.8 ~ -2.8 |
| Pixel size (Å) | 0.83 |
| Movies collected | 5152 |
| Symmetry imposed | C1 |
| Final particle images (no.) | 340,080 |
| Map resolution (Å) | 3.1 |
| FSC cutoff | 0.143 |
| Map resolution range | 2.9 ~ 4.9 |
| Sharpening B-factor (Å2) | -105.5 |
| Software used to process data | cryoSPARC |
| | |
| Refinement statistics | |
| Initial model used | 6P6I |
| Model resolution (FSC = 0.5) | 2.9 |
| Correlation Coefficient (Mask) | 0.83 |
| Number of protein atoms (non-H) Residues | 8970 1150 |
| R.m.s. deviations | |
| Bonds (Å) | 0.007 |
| Bond angles (º) | 0.781 |
| | |
| Validation | |
| MolProbity score | 2.26 |
| Clash score | 35.21 |
| Poor rotamers (%) | 0.00 |
| | |
| Ramachandran plot | |
| Favored (%) | 96.48 |
| Allowed (%) | 3.43 |
| Disallowed (%) | 0.09 |
| | |
| EMDB access code | EMD-43498 |
| PDB access code | 8VSI |

## Slide 8
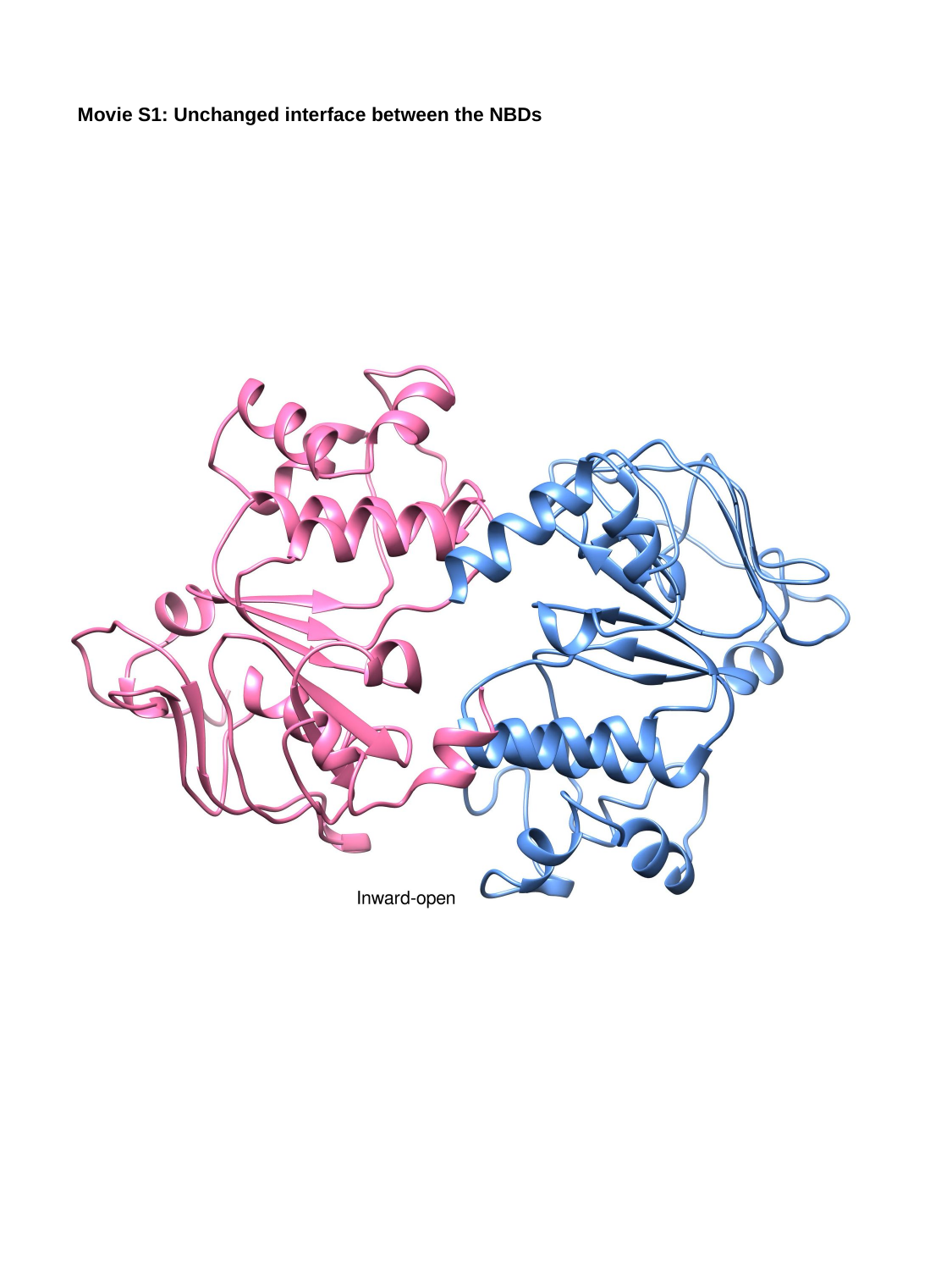

Movie S1: Unchanged interface between the NBDs

## Slide 9
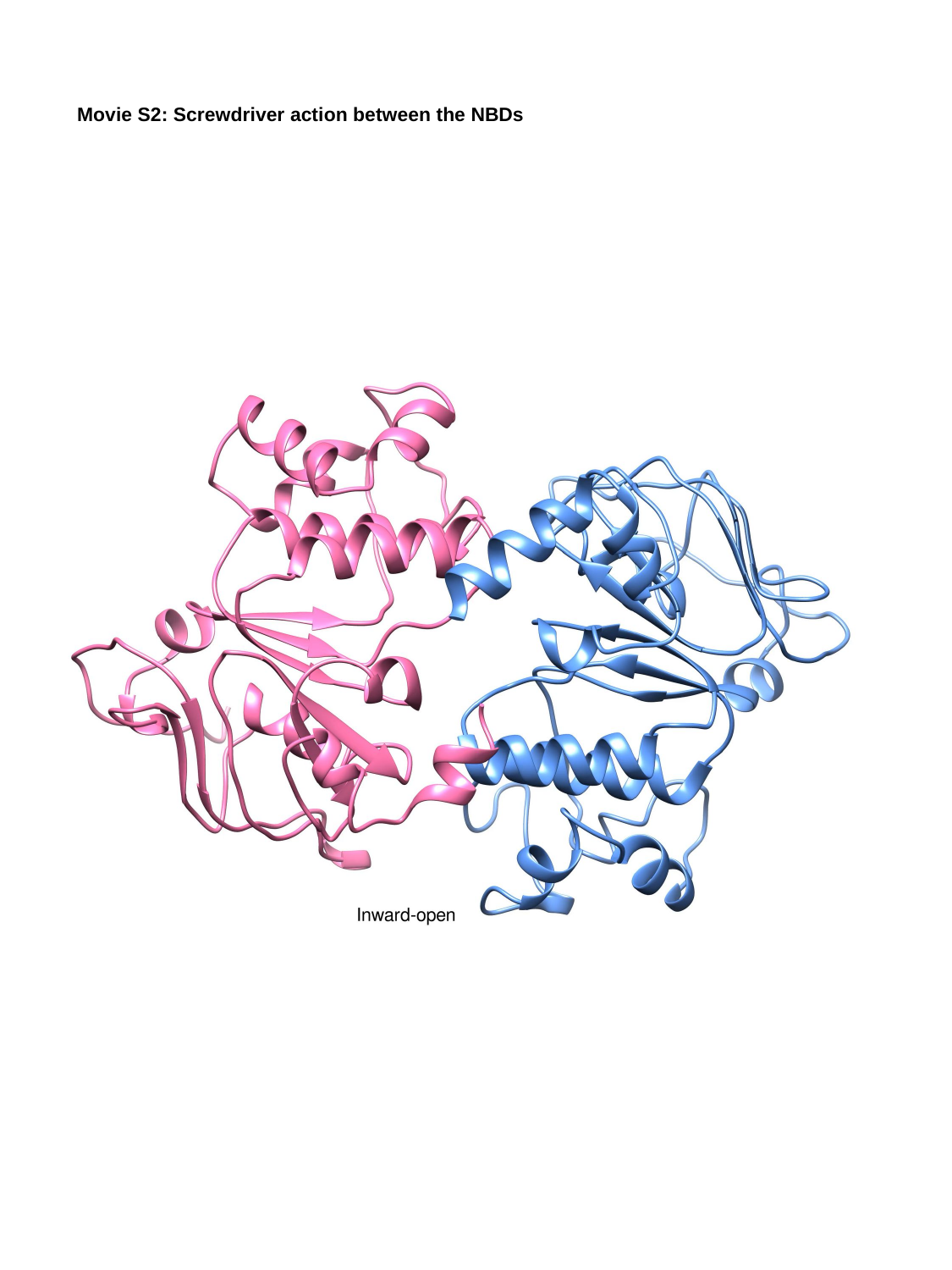

Movie S2: Screwdriver action between the NBDs
